# Single and multiple resistance QTL delay symptom appearance and slow down root colonization by *Aphanomyces euteiches* in pea near isogenic lines

**DOI:** 10.1186/s12870-016-0822-4

**Published:** 2016-07-27

**Authors:** C. Lavaud, M. Baviere, G. Le Roy, M. R. Hervé, A. Moussart, R. Delourme, M-L. Pilet-Nayel

**Affiliations:** 1 INRA, UMR IGEPP 1349, Institut de Génétique, Environnement et Protection des Plantes, Domaine de la Motte au Vicomte, BP 35327, 35653 Le Rheu cedex, France; 2 PISOM, UMT INRA/Terres Inovia, UMR IGEPP 1349, Domaine de la Motte au Vicomte, BP 35327, 35653 Le Rheu cedex, France; 3 Terres Inovia, 11 rue de Monceau, CS 60003, 75378 Paris cedex 08, France

**Keywords:** Near Isogenic Lines (NILs), Partial resistance, *Pisum sativum*, Q-PCR, Quantitative Trait Loci (QTL), Root colonization speed, Root rot, Symptom appearance

## Abstract

**Background:**

Understanding the effects of resistance QTL on pathogen development cycle is an important issue for the creation of QTL combination strategies to durably increase disease resistance in plants. The oomycete pathogen *Aphanomyces euteiches*, causing root rot disease, is one of the major factors limiting the pea crop in the main producing countries. No commercial resistant varieties are currently available in Europe. Resistance alleles at seven main QTL were recently identified and introgressed into pea agronomic lines, resulting in the creation of Near Isogenic Lines (NILs) at the QTL. This study aimed to determine the effect of main *A. euteiches* resistance QTL in NILs on different steps of the pathogen life cycle.

**Results:**

NILs carrying resistance alleles at main QTL in susceptible genetic backgrounds were evaluated in a destructive test under controlled conditions. The development of root rot disease severity and pathogen DNA levels in the roots was measured during ten days after inoculation. Significant effects of several resistance alleles at the two major QTL *Ae-Ps7.6* and *Ae-Ps4.5* were observed on symptom appearance and root colonization by *A. euteiches*. Some resistance alleles at three other minor-effect QTL (*Ae-Ps2.2*, *Ae-Ps3.1* and *Ae-Ps5.1*) significantly decreased root colonization. The combination of resistance alleles at two or three QTL including the major QTL *Ae-Ps7.6* (*Ae-Ps5.1*/*Ae-Ps7.6* or *Ae-Ps2.2*/*Ae-Ps3.1*/*Ae-Ps7.6*) had an increased effect on delaying symptom appearance and/or slowing down root colonization by *A. euteiches* and on plant resistance levels, compared to the effects of individual or no resistance alleles.

**Conclusions:**

This study demonstrated the effects of single or multiple resistance QTL on delaying symptom appearance and/or slowing down colonization by *A. euteiches* in pea roots, using original plant material and a precise pathogen quantification method. Our findings suggest that single resistance QTL can act on multiple or specific steps of the disease development cycle and that their actions could be pyramided to increase partial resistance in future pea varieties. Further studies are needed to investigate QTL effects on different steps of the pathogen life cycle, as well as the efficiency and durability of pyramiding strategies using QTL which appear to act on the same stage of the pathogen cycle.

**Electronic supplementary material:**

The online version of this article (doi:10.1186/s12870-016-0822-4) contains supplementary material, which is available to authorized users.

## Background

Genetic resistance is a major approach for sustainable plant disease management. Although polygenic partial resistance is considered more durable than monogenic complete resistance, little is known about the mechanisms involved in this type of resistance [1, 2]. Only a few studies identified genes underlying resistance quantitative trait loci (QTL) [2–4]. These studies suggested that a large diversity of gene functions is involved in polygenic plant resistance [5]. This diversity in resistance QTL mechanisms suggests that resistance QTL target various steps in the pathogen life cycle, and, indeed, partial resistance has been reported to act on different stages of pathogen development. Pyramiding of resistance QTL targeting different steps in the pathogen life cycle would have a better chance of blocking disease development and should increase resistance levels. It may also make it more difficult for pathogens to adapt and thus be a way to improve the potential for resistance durability [6–8]. Few studies have identified the pathogen life history traits or development cycle steps that resistance QTL target, which is all the more complicated since QTL are difficult to mendelize and individually attribute to a phenotype. The approaches used in previous studies included QTL detection in bi-parental populations for plant resistance at specific steps of the pathogen life cycle and evaluation of Near-Isogenic Lines (NILs) differing from each other for resistance QTL introgressed into susceptible genetic backgrounds [9, 10]. Using NILs, two QTL were shown to act specifically on different stages of *Setosphaeria turcica* cycle (leaf penetration and colonization) in maize [7]. In contrast, in the *Puccinia striiformis*/barley interaction, three QTL were reported to act individually on several components of resistance (latent period, infection efficiency, lesion size and pustule density; [11]).

Aphanomyces root rot, caused by the soilborne oomycete *Aphanomyces euteiches* Drechs. [12], is a major limitation of pea, cultivated for its protein content and ability to fix atmospheric nitrogen. The disease causes translucent lesions on the rootlets, which evolve into brown rot affecting the entire root system and epicotyl [13]. Above ground, the disease causes yellow leaves and even death. Two main *A. euteiches* pathotypes have been reported in pea [14]. Pathotype I is dominant in Europe and was observed in the United States. Pathotype III is specific to some locations in the United States (Onfroy C, Tivoli B, Grünwald NJ, Pilet-Nayel ML, Baranger A, Andrivon D, Moussart A: Aggressiveness and virulence of *Aphanomyces euteiches* isolates recovered from pea nurseries in the United States and France, submitted). The primary inoculum consists of oospores that can persist for up to 10 years in the soil [15]. Epidemics spread primarily from the rapid dispersal of bi-flagellate mobile zoospores released by germinated oospores in the soil. When host root or root exudates are detected, zoospores germinate and penetrate into the roots. The roots are then colonized by mycelium which differentiates into haploid antheridia and oogonia. After sexual reproduction, new diploid oospores are produced. Long-distance dissemination of oospores is mediated by transportation of contaminated soil or materials, or infected plants. In pea plots, crop losses can reach 100 % in disease favorable conditions [16]. Currently the only method of disease management is a soil inoculum potential test to avoid highly infested fields [17].

The generation of *A. euteiches* resistant pea varieties is thus a key factor in integrated strategies for root rot management. However, pea sources of resistance are scarce [18], polygenically inherited and provide only partial resistance levels [19, 20]. Recombinant inbred lines (RILs) populations, obtained from four partially resistant lines, were used for the detection of resistance QTL [21–24]. Meta-QTL analysis identified 23 genomic regions associated with pea resistance to *A. euteiches*, including seven main resistance QTL among which two were major-effect QTL [19]. These QTL were detected consistently across various environments (years, localizations), *A. euteiches* strains and for different scoring criteria. Each of the seven QTL was detected in at least two RIL populations, suggesting a common genetic basis between partial resistance sources. A Marker-Assisted Backcrossing (MAB) program was then conducted to obtain NILs carrying resistance alleles at zero, one, two or three of the seven QTL in several pea lines [25]. Evaluation of the NILs for resistance to *A. euteiches* in controlled conditions allowed major-effect and some minor-effect QTL to be validated, based on a routinely used disease severity test [13, 25, 26]. However, no knowledge is available about the effect of the main resistance QTL on the disease development or *A. euteiches* life cycle, which could be used to support recommendations in QTL pyramiding strategies to durably increase partial resistance. Kraft and Boge [27] reported that partial resistance to *A. euteiches* in pea breeding lines and germplasm was associated with reduced oospore production, pathogen multiplication, zoospore germination and slower lesion development. The genetic components of partial resistance targeting these pathogen life cycle steps were not identified. Precise *A. euteiches* quantification methods were developed for finer evaluation of pea resistance during pathogen development, including enzyme-linked immunosorbent assays (ELISA; [27, 28]), specific fatty acids analysis [29] or, more recently, *A. euteiches* DNA quantification using Quantitative-PCR (Q-PCR; [30, 31]). Due to its high sensitivity, specificity and reproducibility, Q-PCR is an ideal method for detecting minor changes in host resistance [32] and commonly used for resistance evaluation in different pathosystems [1, 7].

The aim of this study was to identify the effect of the main resistance QTL, individually or in combination, on two major steps of the disease development cycle, including symptom appearance and root colonization. A selection of previously developed NILs [25], carrying individual or selected combinations of resistance QTL, were evaluated over time in destructive kinetic assays. The root rot disease severity and quantity of *A. euteiches* DNA in the plant were assessed in inoculated plants under controlled conditions. The effects of each QTL in limiting *A. euteiches* disease development were attributed to one or two of the steps studied. Effects of combined compared to individual QTL were analyzed and used to determine whether pyramided QTL with similar or different individual effects could enhance partial resistance levels.

## Methods

### Plant and pathogen material

Among the 157 NILs produced at INRA, UMR IGEPP and described by Lavaud et al. [25], a total of 23 NILs was used in this study, each carrying or not resistance allele(s) at the main *A. euteiches* resistance QTL in the susceptible reference genetic backgrounds used for QTL detection (Table 1). The lines included were: i) Three NILs carrying no resistance alleles, i.e. with similar genomes to the recipient lines, as susceptible controls. ii) Twelve NILs, each carrying a resistance allele at one QTL, for testing individual QTL effects. The twelve NILs represented all the introgressions targeted in the previous MAB scheme. iii) Eight NILs carrying resistance alleles at two or three QTL for testing effects of selected QTL combinations, depending on individual QTL results. Based on genotyping data, each NIL with zero or one QTL was chosen among sister NILs [25] as that with a better return to the recipient genome outside the QTL and a smaller heterozygosity level (data not shown). All available sister NILs with the chosen combinations of two or three QTL were kept in this study.

**Table 1:** Description of the NILs and controls used

NIL^a^	Donor line^b^	Recipient line^c^	Generation	Introgressed QTL^d^	Experiment
0-QTL					
NIL1-0b	RIL 831.08	Puget	BC_5_F_3_	-	2
NIL4-0b	RIL 847.50	DSP	BC_5_F_3_	-	1,3
NIL7-0b	RIL BAP8.70	Baccara	BC_5_F_3_	-	1,4
Single-QTL					
NIL1-4.5b	RIL 831.08	Puget	BC_5_F_3_	*Ae-Ps4.5*	2
NIL4-5.1a	RIL 847.50	DSP	BC_5_F_3_	*Ae-Ps5.1*	3
NIL4-5.1b	RIL 847.50	DSP	BC_5_F_3_	*Ae-Ps5.1*	1
NIL4-7.6a	RIL 847.50	DSP	BC_5_F_3_	*Ae-Ps7.6*	1,3
NIL7-4.1a	RIL BAP8.70	Baccara	BC_5_F_3_	*Ae-Ps4.1*	1
NIL7-7.6a	RIL BAP8.70	Baccara	BC_5_F_3_	*Ae-Ps7.6*	1
NIL10-1.2a	RIL BAP8.195	Baccara	BC_5_F_3/4_	*Ae-Ps1.2*	1
NIL10-2.2c	RIL BAP8.195	Baccara	BC_5_F_3_	*Ae-Ps2.2*	1
NIL10-3.1b	RIL BAP8.195	Baccara	BC_5_F_3_	*Ae-Ps3.1*	1
NIL13-2.2a	552	Baccara	BC_6_F_4_	*Ae-Ps2.2*	1,4
NIL13-3.1a	552	Baccara	BC_6_F_3/4_	*Ae-Ps3.1*	1,4
NIL13-7.6b	552	Baccara	BC_6_F_4_	*Ae-Ps7.6*	1,4
QTL combination					
NIL4-5.1/7.6a	RIL 847.50	DSP	BC_5_F_3_	*Ae-Ps5.1 + Ae-Ps7.6*	3
NIL4-5.1/7.6b	RIL 847.50	DSP	BC_5_F_3/4_	*Ae-Ps5.1 + Ae-Ps7.6*	3
NIL13-2.2/7.6a	552	Baccara	BC_6_F_3_	*Ae-Ps2.2 + Ae-Ps7.6*	4
NIL13-2.2/7.6b	552	Baccara	BC_6_F_3_	*Ae-Ps2.2 + Ae-Ps7.6*	4
NIL13-3.1/7.6a	552	Baccara	BC_6_F_4_	*Ae-Ps3.1 + Ae-Ps7.6*	4
NIL13-3.1/7.6b	552	Baccara	BC_6_F_4_	*Ae-Ps3.1 + Ae-Ps7.6*	4
NIL13-2.2/3.1/7.6a	552	Baccara	BC_6_F_3_	*Ae-Ps2.2 + Ae-Ps3.1 + Ae-Ps7.6*	4
NIL13-2.2/3.1/7.6b	552	Baccara	BC_6_F_4_	*Ae-Ps2.2 + Ae-Ps3.1 + Ae-Ps7.6*	4
Control lines					
90-2131	-	-	-	-	1,3
PI180693	-	-	-	-	1
552	-	-	-	-	1,4
RIL831.08	-	-	-	-	2
RIL847.50	-	-	-	-	3

NILs carrying resistance alleles at zero, one, two or three of the seven *A. euteiches* resistance QTL in pea [25]

^a^NILs are coded as follow: NIL“NIL set number”-“0 for zero QTL or QTL number(s)” “sister NIL letter (a or b)” [25]. ^b^Lines used as donors of resistance alleles at one to three QTL in the Marker-Assisted Backcrossing (MAB) scheme [25]. ^c^Susceptible parents of the RIL populations in which the QTL were detected, used as reference recipient parents in the MAB scheme [19]. ^d^QTL introgressed from the donor lines in the MAB scheme [25]

Five parental lines of NILs and/or RILs previously used for QTL detection were included as partial resistance controls in the experiments [25] (Table 1). Resistance conferred by the NIL parental lines RIL 831.08, RIL 847.50, as well as RIL BAP8.70 and RIL BAP8.195, was derived from the breeding or germplasm lines 90–2079, 90–2131 and PI180693, respectively, used as parents of the RIL populations previously studied [19]. Pathogen material included the *A. euteiches* strains RB84 and Ae109, belonging to pea pathotypes I and III, respectively. The two strains were both previously used for NILs resistance evaluation in Lavaud et al. [25].

### Disease experiments under controlled conditions

Two experiments were performed under controlled conditions to study the effects of individual QTL on resistance to each of the two strains, respectively. Experiment #1 included two and ten NILs carrying zero and one resistance QTL, respectively, as well as three resistant controls (Table 1) and was inoculated with the RB84 strain. Experiment #2 included the NIL pair with or without the major-effect resistance allele to pathotype III at QTL *Ae-Ps4.5* [25] as well as the resistant control RIL 831.08 (Table 1), and was inoculated with the Ae109 strain. Two other experiments were conducted to evaluate the effects of a selection of QTL combinations on resistance to the RB84 strain. Experiment #3 and #4 included two NILs carrying two resistance QTL from the DSP x RIL 847.50 cross and six NILs carrying two or three resistance QTL from the Baccara x 552 cross, respectively. Both experiments also included the corresponding zero and single QTL NILs and resistance allele donors, as controls (Table 1).

Each experiment included two biological replicates. In each biological replicate, all the lines were evaluated in a randomized complete block design with four blocks and five plants per line in a pot in each block. The plants were harvested at each of the seven time points studied after inoculation in a destructive test. Disease resistance tests were carried out in a growth chamber on seven-day old seedlings grown in vermiculite and inoculated with a 200 zoospores per ml inoculum of pure culture strain, as described in Lavaud et al. [25]. The tests were performed at 20 °C for 16 h of day and 18 °C for 8 h of night. The Disease Severity (DS) was scored on each seedling at two, three, four, five, six, seven and ten days after inoculation, on the different plants of each line at each scoring day, using a 0 (no symptoms) to 5 (dead plant) scoring scale as in Lavaud et al. [25]. In each biological replicate, all the vermiculite was removed from plant roots. Two tissue samples from bulked five plant roots from two blocks were retained for DNA extraction and *A. euteiches* DNA quantification (i.e.*,* a total of four Q-PCR blocks over the two biological replicates for each experiment). At each scoring day, roots were harvested by cutting at the seed level. Roots from the five plants of a pot were then pooled in 50 ml Sarstedt conical tubes containing 5 ml of 3 mm glass beads for grinding later and placed at −80 °C for at least 24 h. Root samples were then lyophilized (3 days, −24 °C) and ground (9 min). Ten mg of powdered roots was placed in each well of a 96-well plate. Each plate also included two empty wells as negative controls. DNA was extracted with an automated DNA extraction robot oKtopure® (LGC Genomics, Germany) and DNA concentrations were normalized at 20 ng/μl.

### Quantitative PCR (Q-PCR) for *A. euteiches* DNA quantification

Q-PCR reactions were performed using the primer/probe set 136F-161T-211R amplifying a 76-bp fragment specific to *A. euteiches,* developed by Vandemark et al. [31]. Primers were synthesized by Sigma Life Science (USA). The probe was labeled with 6-FAM (6-carboxyfluorescein) at its 5’ -terminus and with MGB-NFQ (Minor Groove Binder - Non Fluorescent Quencher) at its 3’ -terminus (Applied Biosystems®, USA). Optimal primer/probe concentrations and amplification cycling conditions were determined in optimization tests. For each DNA sample, triplicate reactions were run in 20 μl reactions containing 40 ng of DNA, 400 nM primer 136F, 400 nM primer 211R, 200 nM probe 161T, 2 μl of ddH_2_O and 10 μl of 2X TaqMan® Universal Master Mix II (Applied Biosystems®, USA). Amplification and detection of fluorescence were carried out on a LightCycler® 480 Instrument II real-time PCR system (Roche Life Science, Germany). PCR reactions consisted of a cycle at 95 °C for 10 min, followed by 50 cycles at 95 °C for 15 s and 60 °C for 45 s. Each analysis also included three control reactions, in which DNA was substituted for ddH_2_O. The amount of *A. euteiches* DNA was estimated using a calibration scale (10^2^, 10^3^, 10^4^, 10^5^, 10^6^, 10^7^ and 10^8^ copies of the 76-bp fragment of *A. euteiches* RB84 strain purified DNA; 3 technical replicates). Q-PCR data were analyzed with LightCycler® 480 software 1.5 (Roche Life Science, Germany) using the same parameters for all reactions. For each Q-PCR reaction, the threshold cycle number (CT), which corresponds to the PCR cycle number at which the fluorescence signal exceeds the detection threshold, was plotted against each of the log10 for the 76-bp fragment copy number. The reaction efficiency (E) was calculated as follow: *E = (10^(−1/b))-1* with *b* corresponding to the slope of the linear regression equation of standard curves. Samples with CT standard deviation values between technical replicates exceeding 0.5 were removed from the analysis.

### Data analysis

Statistical analyses were performed using R software version 3.1.2 [33].

For each experiment and scoring day, correlations between biological replicate and block data, were assessed on genotype means of DS scores and DNA quantification values, using Pearson coefficients (α = 5 %). Correlations between data from the different experiments were also estimated based on common lines, from genotype mean values over all the blocks in the two biological replicates. Statistical analysis of DS scores and DNA quantification data was performed for each experiment and scoring day. Analysis was also performed for three variables derived from DS and DNA amount values, including (i) the probability of symptom appearance corresponding to the percentage of plants with symptoms at each scoring day, (ii) the root colonization speed, corresponding to the slope of the curve of the changing amount of DNA from the scoring day at which more than 10,000 copies of pathogen DNA were detected and (iii) the area under the curve progression of the disease (AUDPC), calculated from the DNA quantification values of the pathogen, according to the formula proposed by Shaner and Finney [34]. The pea line values for these three variables were calculated in each block and experiment.

DS scores, as ordinal qualitative data, were analyzed using a cumulative linked mixed model [CLMM; ‘clmm’ function, ‘ordinal’ package [35, 36]]. DNA quantification values, root colonization speed and AUDPC, as quantitative data, were analyzed using a linear mixed model [LMM; ‘lmer’ function, ‘lme4’ package [37]]. Probability of symptom appearance values were analyzed using a Generalized Linear Mixed Model (GLMM; ‘glmer’ function, ‘lme4’ package [37]).

For each model, we considered the genotype as fixed factor, the blocks of the two biological replicates as random factors. A Likelihood Ratio test (LR) (α = 5 %) and a Wald test (α = 5 %) were applied for evaluating the genotypic effect in the CLMM model and in the GLMM or LMM model, respectively.

Least Square Means (LSMeans) were estimated for all the variables on each genotype, using the ‘lsmeans’ function of the ‘lsmeans’ package [38]. For the DS variable, LSMeans were calculated on the scale of the latent variable implied by the CLMM, for each genotype and day [39, 40]. For each variable, multiple comparisons of LSMeans between genotypes of each NIL set were performed with the Tukey test (α = 5 %), using the ‘cld’ function of the ‘lsmeans’ package.

## Results

### Effect of Single resistance QTL on *A. euteiches* development

#### Disease severity scores

DS scores for all the lines were significantly correlated between the two biological replicates at each scoring date in experiments #1 and #2 (*r* > 0.82, *P* < 0.05 and *r* > 0.90 *P* < 0.05, respectively), except for one replicate on the fourth day in experiment #2, which was removed from the analysis.

A total of five NILs carrying single QTL showed significant differences in LSMeans scores compared to their control NIL without the QTL, for at least one scoring day (Table 2, Additional file 1A). In experiment #1, the level of partial resistance to the RB84 strain was significantly higher (*P* < 0.001) in all the NILs carrying the single QTL *AePs7.6* from the different donors (NIL4-7.6a, NIL7-7.6a and NIL13-7.6b), compared to their corresponding control NILs without the QTL (NIL4-0b or NIL7-0b). NIL4-5.1b, carrying the single QTL *Ae-Ps5.1*, showed significantly lower DS values than the ones of control NIL only at the fourth day after inoculation (*P* < 0.05). In experiment #2, significantly lower (*P* < 0.001) DS scores were obtained for the NILs carrying the major QTL *Ae-Ps4.5* (NIL1-4.5b) compared to the control NIL, from the sixth to tenth day, for the Ae109 strain (Table 2, Additional file 1A). Higher levels of partial resistance in the resistant donor lines were confirmed to be significant between five and ten days after inoculation in each NIL set compared to each susceptible control NIL (*P* < 0.001), except for 90–2131 (Table 2).

**Table 2:** Disease Severity lsmeans scores in single-QTL NIL experiments

Experiment	Cross^a^	Genotype	QTL^b^	Day after inoculation^c^						
(Strain)				2^d^	3^d^	4^e^	5	6	7	10
1	DSP * RIL 847.50	NIL4-0b	*-*	NA	NA	2,0	−1,6	−2,3	−4,1	−3,3
(RB84)		NIL4-5.1b	*Ae-Ps5.1*	NA	NA	3,9*	−0,5	−1,0	−3,7	−2,4
		NIL4-7.6a	*Ae-Ps7.6*	NA	NA	4,1*	1,7***	0,4***	−0,8***	−0,6***
		90-2131	*-*	NA	NA	3,1	−1,3	−1,1	−4,2	−2,9
	Baccara * RIL BAP8.70	NIL7-0b	*-*	NA	NA	2,6	−2,5	−3,7	−1,0	−1,9
		NIL7-4.1a	*Ae-Ps4.1*	NA	NA	2,0	−2,6	−3,7	−0,7	−2,2
		NIL7-7.6a	*Ae-Ps7.6*	NA	NA	4,0	1,6***	−0,1***	1,4**	0,2**
		PI180693	*-*	NA	NA	4,5	1,4***	0,9***	3,0***	2,3***
	Baccara * RIL BAP8.195	NIL7-0b	*-*	NA	NA	0,8	−2,4	−3,2	−1,0	−2,3
		NIL10-1.2a	*Ae-Ps1.2*	NA	NA	1,3	−1,4	−2,8	−1,6	−3,2
		NIL10-2.2c	*Ae-Ps2.2*	NA	NA	0,7	−2,6	−2,5	−0,5	−1,9
		NIL10-3.1b	*Ae-Ps3.1*	NA	NA	1,1	−2,4	−3,0	−0,8	−2,3
		PI180693	*-*	NA	NA	2,6	1,3***	0,7***	2,8***	2,5***
	Baccara * 552	NIL7-0b	*-*	NA	NA	0,8	−1,9	−3,9	−1,7	−2,9
		NIL13-2.2a	*Ae-Ps2.2*	NA	NA	0,5	−1,2	−2,7	−1,9	−3,4
		NIL13-3.1a	*Ae-Ps3.1*	NA	NA	2,6	−1,1	−2,8	−1,1	−1,9
		NIL13-7.6b	*Ae-Ps7.6*	NA	NA	1,9	0,1*	−0,3***	−0,3	−2,2
		552	*-*	NA	NA	2,6	1,2***	0,4***	0,6**	0,0**
2	Puget * RIL 831.08	NIL1-0b	*-*	NA	NA	1,6	1,6	−0,9***	−2,2	−2,7
(Ae109)		NIL1-4.5b	*Ae-Ps4.5*	NA	NA	3,6	2,5	2,1	1,4***	1,2***
		RIL 831.08	*-*	NA	NA	3,7	4,8***	2,6***	1,6	1,6***

^a^“Recipient x donor” cross lines from which each NIL was produced in the previous MAB scheme [25]. ^b^QTL introgressed in each NIL from the previous MAB scheme [25]. ^c^LSMeans disease severity (DS) scores obtained on each genotype and scoring day from the CLMM analysis of each NIL set in experiments #1 and #2. Lsmeans scores were obtained from the DS score probabilities for each genotype and scoring day represented in Additional file 1A. LSMeans DS values ranged from −4.2 to 4.8, according to the scale of the latent variable implied by the CLMM. Significant differences between LSMeans values of the single-QTL NILs or the resistant control, and the control-NIL without QTL are indicated by *(0.01 < P < 0.05), **(0.001 < *P* < 0.01) and ***(*P* < 0.001). ^d^At two and three days after inoculation, LSMeans DS scores could not be estimated from CLMM since some lines did not have symptoms. ^e^At four days after inoculation, LSMeans DS scores were estimated from data obtained in one biological replicate in experiment #2

#### Quantification of A. euteiches DNA

The efficiency (E) of the Q-PCR reactions ranged from 93.6 % to 100.1 % for the 16 plates. These results are in agreement with Schena et al. [41] who recommended efficiencies as close as possible to 100 %. Two days after inoculation, DNA amount values could not be considered for all the lines due to the small amount of pathogen DNA in the roots and the limit of detection of 1,000 copies. Similarly, the amount of DNA could not be quantified for 552, RIL 831.08 and NIL1-4.5b before four, five and six days after inoculation, respectively (Table 3). Six percent of the block data for DNA quantity was removed from the analysis because of CT standard deviations exceeding 0.5 between technical triplicates in a block. The amount of pathogen DNA in all the lines correlated significantly between the two biological replicates at each scoring date in experiments #1 and #2 (*r* > 0.95, *P* < 0.05 and *r* > 0.73 *P* < 0.06, respectively), except at the fourth day in experiments #1 and #2, for which one replicate was removed from the analysis.

**Table 3:** *A. euteiches* DNA amounts in roots in single-QTL NIL experiments

Experiment (Strain)	Cross^a^	Genotype	QTL^b^	Day after inoculation^c^						
				2	3	4^d^	5	6	7	10
1	DSP * RIL 847.50	NIL4-0b	*-*	NA	3,0 ± 0,5	44,9 ± 4,3	201,8 ± 21,7	387,0 ± 35,0	576,8 ± 46,8	1110,8 ± 69,2
(RB84)		NIL4-5.1b	*Ae-Ps5.1*	NA	2,5 ± 0,5	11,2 ± 4,3***	117,8 ± 21,7***	325,4 ± 33,5*	425,7 ± 45,7***	839,4 ± 69,2***
		NIL4-7.6a	*Ae-Ps7.6*	NA	2,0 ± 0,5	15,5 ± 4,3***	81,6 ± 22,6***	155,4 ± 35,0***	351,9 ± 45,3***	699,5 ± 70,6***
		90-2131	*-*	NA	5,3 ± 0,4	31,6 ± 4,3*	162,0 ± 21,7*	297,7 ± 34,8**	348,5 ± 45,3***	858,8 ± 69,2**
	Baccara * RIL BAP8.70	NIL7-0b	*-*	NA	5,3 ± 0,3	43,9 ± 4,0	294,9 ± 16,6	523,2 ± 17,1	679,7 ± 29,1	1721,5 ± 80,9
		NIL7-4.1a	*Ae-Ps4.1*	NA	6,0 ± 0,3	67,4 ± 4,0	260,0 ± 16,6	421,9 ± 17,1**	650,2 ± 29,1	1478,2 ± 80,9
		NIL7-7.6a	*Ae-Ps7.6*	NA	1,6 ± 0,6***	17,0 ± 4,0***	58,8 ± 16,6***	251,0 ± 17,1***	437,4 ± 26,1***	882,4 ± 80,9***
		PI180693	*-*	NA	2,0 ± 0,5***	17,3 ± 4,0***	53,7 ± 16,6***	81,6 ± 26,4***	167,5 ± 26,1***	401,7 ± 80,9***
	Baccara * RIL BAP8.195	NIL7-0b	*-*	NA	5,3 ± 0,8	43,9 ± 12,3	294,9 ± 22,5	523,2 ± 23,8	652,1 ± 73,9	1721,5 ± 127,2
		NIL10-1.2a	*Ae-Ps1.2*	NA	4,4 ± 0,8	43,3 ± 12,3	180,9 ± 22,5***	362,0 ± 23,8***	827,9 ± 67,0	1450,7 ± 127,2
		NIL10-2.2c	*Ae-Ps2.2*	NA	3,3 ± 0,9*	48,1 ± 12,3	229,4 ± 22,5**	407,7 ± 23,8**	527,7 ± 73,9	1360,2 ± 127,2*
		NIL10-3.1b	*Ae-Ps3.1*	NA	5,6 ± 0,8	47,8 ± 12,3	177,5 ± 22,5***	377,2 ± 27,6***	652,5 ± 73,9	1478,4 ± 127,2
		PI180693	*-*	NA	0,9 ± 1,0***	17,3 ± 12,3***	53,7 ± 22,5***	88,1 ± 33,8***	167,5 ± 67,0***	401,7 ± 127,2***
	Baccara * 552	NIL7-0b	*-*	NA	5,3 ± 0,6	43,9 ± 7,0	294,9 ± 19,6	523,2 ± 21,4	694,8 ± 36,6	1721,5 ± 116,2
		NIL13-2.2a	*Ae-Ps2.2*	NA	6,0 ± 0,6	77,5 ± 7,0	186,8 ± 21,5***	344,9 ± 21,4***	549,2 ± 32,9**	1117,6 ± 143,0**
		NIL13-3.1a	*Ae-Ps3.1*	NA	5,1 ± 0,6	43,7 ± 7,0	168,4 ± 19,6***	247,7 ± 21,4***	451,5 ± 32,9***	991,5 ± 116,2***
		NIL13-7.6b	*Ae-Ps7.6*	NA	1,7 ± 0,6***	19,9 ± 7,0*	76,9 ± 19,6***	178,9 ± 29,7***	341,5 ± 38,1***	681,7 ± 116,2***
		552	*-*	NA	NA	4,2 ± 8,3***	14,0 ± 19,6***	82,8 ± 21,4***	177,0 ± 32,9***	428,6 ± 116,2***
2	Puget * RIL 831 .08	NIL1-0b	*-*	NA	2,6 ± 0,4	32,6 ± 7,7	91,8 ± 26,3	293,5 ± 7,9	535,0 ± 35,0	1349,9 ± 158,7
(Ae109)		NIL1-4.5b	*Ae-Ps4.5*	NA	NA	NA	NA	7,3 ± 7,6***	27,8 ± 35,0***	88,2 ± 158,7***
		RIL 831.08	*-*	NA	NA	NA	30,6 ± 27,1**	26,4 ± 9,1***	92,1 ± 39,6***	304,5 ± 166,6***

^a^“Recipient x donor” cross lines from which each NIL was produced in the previous MAB scheme [25]. ^b^QTL introgressed in each NIL from the previous MAB scheme [25]. ^c^Pathogen DNA amount were obtained on each genotype and scoring day from the LMM analysis of each set of lines in experiments #1 and #2. LSMeans and standard errors of pathogen DNA amount are presented in thousand DNA copies (10^^3^). In each set of lines, significant differences of LSMeans DNA amount between the single-QTL NILs or the resistant control, and the control- NIL without QTL are indicated by *(0.01 < *P* < 0.05), **(0.001 < *P* < 0.01) and ***(*P* < 0.001). ^d^In experiments #1 and #2, LSMeans DNA amount were estimated from data obtained in one biological replicate at the fourth day. NA: Not Available data due to copy number <10^3^

All the NILs carrying single QTL showed a significant lower pathogen DNA amount in the roots at the sixth day after inoculation (*P* < 0.05), compared to the control NILs without QTL (Table 3). In experiment #1, all the NILs carrying resistance alleles at the single major QTL *AePs7.6,* regardless of the resistance donor of origin (NIL4-7.6a, NIL7-7.6a and NIL13-7.6b), had the highest and most consistently significant effects to reduce pathogen DNA amounts over five or six scoring days (*P* < 0.05). The NIL carrying the 90–2131 resistance allele at *Ae-Ps5.1*, had a smaller but still significant effect on reducing the amount of pathogen DNA over five time points (*P* < 0.05), compared to the control NIL. NILs with resistance alleles from 552 at *AePs2.2* and *AePs3.1* significantly reduced (*P* < 0.01) the amount of *A. euteiches* DNA over four days, compared to the control NIL. The four remaining NILs (NIL7-4.1b, NIL10-1.2a, NIL10-2.2c and NIL10-3.1b) had smaller and/or less consistently significant effects at one to four scoring days (*P* < 0.05). In experiment #2, the NIL carrying the major resistance QTL *Ae-Ps4.5* (NIL1-4.5b) had a very small quantity of *A. euteiches* in its roots until ten days after inoculation, which was significantly different from the high DNA quantity observed in the NIL control free of the QTL (*P* < 0.001) (Table 3).

#### Disease development variables

**Fig. 1:**
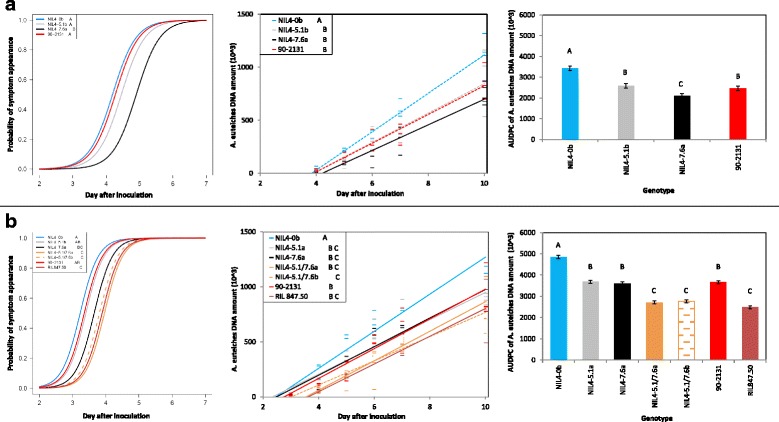
Effects of NILs carrying single or combined resistance QTL *Ae-Ps5.1* and *Ae-Ps7.6* on variables of *Aphanomyces* root rot development cycle. **a**/ Single QTL NIL experiment #1; **b**/ Combined and single QTL NIL experiment #3. The first graph represents the evolution of the probability of symptom appearance for seven days after inoculation, for each line. It corresponds to the percentage of plants with symptoms per block for each scoring day. The second graph shows for each line the root colonization speed, corresponding to the slope of the curve of pathogen DNA amounts per block, until 10 days after inoculation, from 10^4^ DNA copies detected. Pathogen DNA data were used from one biological replicate at the fourth day in experiment #1 and the tenth day in experiment #3. In the third graph, the AUDPC was calculated from the pathogen DNA quantification data over the ten days after inoculation. Bars represent standard errors. Attribution of each line to LSMeans group(s) is indicated by letter(s), according to the Tukey test (*P* < 0.05). Blue and red lines indicate the NIL without QTL and the donor or resistant control lines, respectively

The probability of disease symptom appearance on the NILs was calculated from DS scores data over time, to estimate the effects of resistance QTL in the early stages of the pathogen cycle in the root (pathogen penetration into the roots and development until symptoms appeared). The probability curves obtained for each set of NILs in both experiments (Fig. 1a, Additional file 2) showed that symptoms appeared significantly later for NILs carrying the major resistance QTL *Ae-Ps7.6* (NIL4-7.6a, NIL7-7.6a) or *Ae-Ps4.5* (NIL1-4.5b) than for their corresponding NILs without QTL. The same effect was observed for the donor lines PI180693, 552 and RIL 831.08 compared to the susceptible control NILs.

Two traits were used to measure NIL root colonization by *A. euteiches*: colonization speed and the quantity of pathogen having colonized the roots. The two traits were estimated from the slope and the AUDPC of the pathogen DNA progression curve over ten days after inoculation, respectively. In the two experiments, curves for all the NILs, except three ones (NIL7-4.1a, NIL10-1.2a and NIL10-3.1b), and the four donor lines (90–2131, PI180693, 552 and RIL 831.08), had significantly lower slopes and AUDPC values than control NILs (Fig. 1a and Additional file 2). In particular, the four NILs carrying the single major QTL *Ae-Ps7.6* or *Ae-Ps4.5* had the lowest AUDPC values among all the NILs. Significantly more gentle slopes were observed for NILs with significantly lower DNA levels at more than two scoring days (Table 3), compared to susceptible control NILs.

### Effects of multiple resistance QTL on *A. euteiches* development

Overall, two NIL sets (n°4 and n°13) carrying combinations of QTL which all showed significant individual effects on the symptom appearance and/or root colonization (speed and quantity), were selected for studying multiple QTL effects. In these two NIL sets, combinations of resistance alleles from 90–2131 at *Ae-Ps5.1* and *Ae-Ps7.6*, and from 552 at *Ae-Ps2.2*, *Ae-Ps3.1* and *Ae-Ps7.6*, were tested with the RB84 strain in experiments #3 and #4.

Symptoms developed faster in experiments #3 and #4 than in experiment #1, mainly due to temperature variation between experiments in the growth chamber. A more rapid disease initiation and less discrimination between genotypes was thus observed, especially in experiment #4. However, based on common genotypes, DS scores and *A. euteiches* DNA quantification data were highly correlated between experiments #3 and #1 (*r* > 0.98, *P* < 0.001) as well as #4 and #1 (*r* > 0.84, *P* < 0.001). In both experiments #3 and #4, DS scores and pathogen DNA data were highly correlated between the two biological replicates at each scoring date (*r* > 0.95 *P* < 0.05), except at the fourth day in experiments #3 and #4 for DS scores as well as at the tenth day in experiment #3 and the seventh day in experiment #4 for the amount of pathogen DNA, for which data from one replicate were removed from the analysis. In experiment #3, additional pathogen DNA data were removed from the analysis from one biological replicate for NIL4-7.6a at all days and from the two biological replicates for RIL 847.50 at the 6^th^ day, due to incoherence in data between replicates or days.

In experiment #3, the significant effects of the two single QTL NILs and the RIL 847.50 parental line were confirmed for both DS scores and Q-PCR data, at a later scoring day than in experiment #1 for the NIL with QTL *Ae-Ps5.1* (Table 4, Additional file 1B). The effect of single QTL to delay the symptom appearance probability (*Ae-Ps7.6*) and decrease root colonization (*Ae-Ps7.6* and *Ae-Ps5.1*) was also confirmed. DS scores and the amounts of *A. euteiches* DNA for the two sister NIL carrying both *Ae-Ps5.1* and *Ae-Ps7.6*, and for the RIL 847.50 resistant parental line, were highly significantly reduced (*P* < 0.001) compared to the control NIL at all the scoring days (Table 4). The DS and pathogen DNA values were even significantly reduced from NILs carrying the single QTL *Ae-Ps7.6* at several scoring days (Table 4). The three lines also had significantly increased effects for reducing the symptom appearance probability and the AUDPC compared to the single and/or free QTL NILs and for slowing down root colonization speed compared to the control NIL without QTL (Fig. 1b).

**Table 4:** Disease Severity lsmeans scores and *A. euteiches* DNA amounts in roots, in multiple-QTL NIL experiments

Experiment (Strain)	Cross^a^	Genotype	QTL^b^	Symptom scoring^c^							*A. euteiches* DNA amount^e^						
				Day after inoculation							Day after inoculation						
				2^d^	3^d^	4^f^	5	6	7	10	2	3	4	5	6	7^f^	10^f^
**3**	DSP * RIL 847.50	NIL4-0b	-	NA	NA	−1.6	−2.9	−1.2	−4.6	−5.0	NA	23.8 ± 2.5	226.5 ± 14.1	522.6 ± 23.8	696.1 ± 36.8	817.6 ± 41.7	1142.9 ± 59.4
**(RB84)**		NIL4-5.1a	*Ae-Ps5.1*	NA	NA	−0.7	−1.5	−0.1	−2.4***	−4.6	NA	18.1 ± 2.5	131.8 ± 14.1***	368 ± 21.3***	558.4 ± 33.8**	668.1 ± 35.1*	871.7 ± 59.4***
		NIL4-7.6a^g^	*Ae-Ps7.6*	NA	NA	0.5*	0.0***	1.0**	−1.5	−3.8	NA	11.9 ± 3.1**	98.1 ± 17.2***	339.3 ± 28.1***	561.8 ± 42*	633.1 ± 41.7*	855.8 ± 71.8***
		NIL4-5.1/7.6a	*Ae-Ps5.1* + *Ae-Ps7.6*	NA	NA	1.9***	0.9***	3.5***	0.5***	−0.1***	NA	7 ± 2.5***	73.8 ± 15.2***	**155.7** ± 21.3***	**297.9** ± 33.8***	477.9 ± 31.5***	826.2 ± 59.4***
		NIL4-5.1/7.6b	*Ae-Ps5.1* + *Ae-Ps7.6*	NA	NA	1.2***	0.8***	3.4***	1.3***	−1.4**	NA	9.1 ± 2.5***	71.1 ± 14.1***	**210.6** ± 21.3***	**363.3** ± 36.7***	495.1 ± 31.5***	739.1 ± 59.4***
		90-2131	-	NA	NA	−0.3	−1.7	−0.8	−3.3	−4.3	NA	15.3 ± 2.5*	101.5 ± 14.1***	279.4 ± 21.3***	533.8 ± 33.8***	675.2 ± 35.4	911 ± 59.4***
		RIL 847.50	-	NA	NA	1.2***	1.0***	2.5***	−0.2***	−2.0*	NA	7.6 ± 2.5***	**45.4** ± 14.1***	**170.1** ± 21.3***	NA	**380** ± 35.4***	758.3 ± 59.4***
**4**	Baccara * 552	NIL7-0b	-	NA	NA	0.6	2.0	2.8	5.0	2.7	1.3 ± 0.3	6.9 ± 0.9	70.5 ± 7.9	295.3 ± 38.3	634.7 ± 52.9	806.1 ± 56.1	1948 ± 156.1
**(RB84)**		NIL13-2.2a	*Ae-Ps2.2*	NA	NA	−0.9	0.9	2.3	4.5	2.0	1.6 ± 0.2	12.3 ± 0.9	86.7 ± 7.7	309 ± 36	798.5 ± 50.3	970.4 ± 64.3	2095.5 ± 156.1
		NIL13-3.1a	*Ae-Ps3.1*	NA	NA	−1.3*	0.1*	1.0	3.2	1.7	3.6 ± 0.6	6 ± 1.1	42.5 ± 7.7*	270 ± 35.5	455.6 ± 43.8	761.6 ± 56.1	1384.5 ± 167.9
		NIL13-7.6b	*Ae-Ps7.6*	NA	NA	−2.0***	−0.4***	1.4	2.9*	3.1	NA	3.3 ± 1.3***	28.9 ± 7.7***	239.1 ± 35.5	476.2 ± 53	709.9 ± 56.1	1544.2 ± 156.1
		NIL13-2.2/7.6a	*Ae-Ps2.2* + *Ae-Ps7.6*	NA	NA	−1.2**	0.6	1.4	3.1	3.0	NA	4.7 ± 0.9	55.8 ± 7.9	314.6 ± 35.5	566.9 ± 45.5	805.5 ± 64.3	1573.8 ± 156.1
		NIL13-2.2/7.6b	*Ae-Ps2.2* + *Ae-Ps7.6*	NA	NA	−1.1	0.7	1.8	3.5	2.0	NA	6.8 ± 0.9	66.3 ± 7.9	346.7 ± 37.4	651.1 ± 43.8	901.2 ± 58.3	1416.7 ± 167.9
		NIL13-3.1/7.6a	*Ae-Ps3.1* + *Ae-Ps7.6*	NA	NA	−1.8	−0.8***	0.8	2.3**	1.5	1.1 ± 0.7	3 ± 1***	35.7 ± 7.9**	166.5 ± 36**	431.1 ± 45.5**	694.5 ± 56.1	1254.7 ± 156.1**
		NIL13-3.1/7.6b	*Ae-Ps3.1* + *Ae-Ps7.6*	NA	NA	−2.1	−0.4**	0.9	2.4**	2.5	1.1 ± 0.6	3.4 ± 0.9	46.7 ± 7.9*	259.3 ± 35.5	494 ± 43.8	680 ± 56.1	1522 ± 156.1
		NIL13-2.2/3.1/7.6a	*Ae-Ps2.2* + *Ae-Ps3.1* + *Ae-Ps7.6*	NA	NA	−2.0***	−0.8***	0.9	2.8*	0.9	1.1 ± 0.7	3.1 ± 1.1**	30.9 ± 7.7***	181.8 ± 36*	428.4 ± 43.8*	576.2 ± 64.3*	1231 ± 156.1**
		NIL13-2.2/3.1/7.6b	*Ae-Ps2.2* + *Ae-Ps3.1* + *Ae-Ps7.6*	NA	NA	−1.2**	−0.1*	0.6	3.0	2.9	NA	3.8 ± 0.9**	42.3 ± 7.7*	221.4 ± 36	403.7 ± 43.8**	646.9 ± 64.3	1258.8 ± 161.6*
		552	-	NA	NA	−2.9***	−2.7***	−2.5***	−1.9***	0.3*	NA	1.1 ± 1.5***	3.7 ± 8.5***	**41.4** ± 35.5***	**127.5** ± 43.8***	**286.7** ± 56.1***	**703.7** ± 156.1***

^a^“Recipient x donor” cross lines from which each NIL was produced in the previous MAB scheme [25]. ^b^QTL introgressed in each NIL from the previous MAB scheme [25]. ^c^LSMeans disease severity (DS) scores obtained on each genotype and scoring day from the CLMM analysis of each NIL set in experiments #3 and #4. Lsmeans scores were obtained from the DS score probabilities for each genotype and scoring day represented in Additional file 1B. LSMeans DS values ranged from −5 to 5, according to the scale of the latent variable implied by the CLMM. Significant differences between LSMeans values of the single-QTL NILs or the resistant control, and the control-NIL without QTL are indicated by *(0.01 < *P* < 0.05), **(0.001 < *P* < 0.01) and ***(*P* < 0.001). ^d^At two and three days after inoculation, LSMeans DS scores could not be estimated from CLMM since some lines did not have symptoms. ^e^Pathogen DNA amount were obtained on each genotype and scoring day from the LMM analysis of each NILs set in experiments #3 and #4. LSMeans and standard errors of pathogen DNA amount are presented in thousand DNA copies (10^^3^). In each set of lines, significant differences of LSMeans DNA amount between the single-QTL NILs or the resistant control, and the control- NIL without QTL are indicated by *(0.01 < *P* < 0.05), **(0.001 < *P* < 0.01) and ***(*P* < 0.001). ^f^LSMeans scores were estimated from data in one biological replicate, for DS scoring at the fourth day in experiment #3 and #4 and for pathogen DNA data at the tenth day in experiment #3 and the seventh day in experiment #4. ^g^LSMeans DS scores and pathogen DNA amounts for NIL4-7.6a were estimated from data in one biological replicate at all days. NA: Not Available data due to copy number <10^3^ or to inconsistent data. **In bold**, significant differences of LSMeans DS scores or DNA amounts between the multiple-QTL NILs and the single-QTL NIL NIL4-7.6a for experiment #3 or NIL13-7.6b for experiment #4 (*P* < 0.05)

In experiment #4, the significant effect (*P* < 0.001) of the single QTL NIL carrying QTL *Ae-Ps7.6* was confirmed at earlier scoring days than in experiment #1 for reducing both DS scores and DNA amount, compared to the control NIL without QTL (Table 4, Additional file 1B and 2). The significant effect of the 552 resistant parental line was also confirmed to decrease both variable values at almost all scoring dates (*P* < 0.001) and to reduce values for the three disease development variables estimated, compared to both NILs with the single QTL *Ae-Ps7.6* and without QTL. However in contrast to experiment #1, (i) the single resistance QTL NIL with *Ae-Ps3.1* had a low but significant effect (*P* < 0.05) on reducing DS scores and pathogen DNA amount at only early time points; (ii) the NIL with resistance QTL *Ae-Ps2.2* had more DNA in its roots than the control NIL without QTL from the fifth scoring stage, and thus did not show any significant effect; (iii) the three single QTL NILs did not show significant differences compared to the control NIL for root colonization speed and AUDPC and (iv) the NIL carrying QTL *Ae-Ps7.6* significantly delayed the symptom appearance probability. The two sister NILs carrying the two QTL *Ae-Ps2.2* and *Ae-Ps7.6* showed no effect on decreasing DS scores and *A. euteiches* DNA levels compared to the control NIL without QTL, except the NIL13-2.2/7.6a for DS scores at the fourth day after inoculation. The sister NILs carrying either the two QTL *Ae-Ps3.1* and *Ae-Ps7.6* or the three QTL *Ae-Ps2.2*, *Ae-Ps3.1* and *Ae-Ps7.6*, showed significant effects (*P* < 0.05) on reducing DS scores and pathogen DNA levels at several time points, compared to the NIL without QTL but not compared to the single QTL NIL carrying *Ae-Ps7.6*. Consistently, these bi- or tri-QTL NILs had significantly delayed curves of symptom appearance probability compared to the control NIL without QTL. No bi- or tri-QTL NILs significantly decreased the root colonisation speed. Only the two tri-QTL NILs had lower AUDPC values than the NIL without QTL but not from the NILs carrying single QTL.

## Discussion

This study used NILs as original plant material and Q-PCR as a precise quantification method to study the effects of single and multiple resistance QTL on two steps of Aphanomyces root rot development on pea, symptom appearance and root colonization. Our results demonstrated significant single effects of resistance alleles at two major QTL (*Ae-Ps7.6* and *Ae-Ps4.5)* on the two steps studied and at several minor QTL (*Ae-Ps2.2, Ae-Ps3.1* and *Ae-Ps5.1*) on root colonization. Selected combinations of two or three of the most significant single effect-QTL, including QTL *Ae-Ps7.6,* were subsequently tested. The NILs carrying QTL combinations showed significantly increased or similar effects on delaying symptom appearance and slowing down root colonization by the pathogen compared to single QTL NILs, depending on the experiment. Our findings validate previous QTL effects [25] and also point out the relevance of Q-PCR for accurately quantifying *A. euteiches* in pea roots at distinct stages of fungal pathogenesis.

### Disease severity kinetics and *A. euteiches* DNA quantification allowed two steps of disease development to be evaluated

In this study, we used a kinetic pathology test to measure *Aphanomyces* root rot development in young pea roots for ten days after inoculation. This test was destructive, as plants were uprooted at each scoring day to evaluate disease severity and to sample roots for pathogen DNA quantification. However, for each pea line, scores obtained for different plants at the different scoring days were assumed to be comparable since the NILs were self-pollinated for three or four generations (BC_5/6_F_3/4_) and individuals from each NIL were expected to have identical genomes. Non-destructive methods have been reported for measuring *Aphanomyces* root rot development over time. Although pathogen DNA could not be quantified using these methods, they had the advantage that symptom evolution could be observed on the same plant. The “Rolled Towel assay” developed by Malvick et al. [42] used pre-germinated plants in paper towels placed at 20 °C to measure the evolution of root rot symptoms on the same plants for 21 days. However, secondary disease infections developed in the towels with this method [43]. An in vitro test was also designed to evaluate the development of *Aphanomyces* root rot symptoms on *M. truncatula* roots at three, 15 and/or 21 days after inoculation [44, 45].

Quantitative-PCR is a development of the original PCR technique that allows accurate quantification of a target amplicon based on dye fluorescence included in the reaction. This assay has been commonly used as a rapid and efficient tool for the accurate and specific detection and quantification of pathogens in plants, such as *Sclerospora graminicola* [46] in pear millet, *Fusarium solani* in soybean [47] or *Thielaviopsis basicola* in cotton [48]. It was used by Vandemark et al. [31] to specifically quantify *A. euteiches* DNA, based on a 76 bp amplicon, and successfully applied for pathogen quantification in pea roots even if the quantity of pathogen DNA was not always correlated with disease severity.

In our study, two of the disease development cycle phases, symptom appearance and root colonization, were observed using kinetic pathology test and Q-PCR. Symptom appearance results from the early steps of the pathogen life cycle in the plant. When non-destructive tests are used, these early steps are usually measured by the latent or incubation periods, especially for aerial pathogens. Here, the date at which the first symptoms appeared on the roots, corresponding to the incubation period, was difficult to measure. We would have needed to increase the number of time points observed in the first days after inoculation to, for example, every six hours. We could have also slown down disease development by reducing the test temperature to 15-18 °C for example, to better discriminate between genotypes. Instead, we calculated a probability of symptom appearance based on DS symptoms detected in the roots at different time points, which allowed NILs carrying major effect QTL to be significantly differentiated at the early steps of the interaction. At these early steps, pathogen DNA levels were not used to differentiate NILs since Q-PCR did not reliably detect less than 1,000 copies of the amplified DNA fragment. Pathogen colonization of the plant roots could be accurately measured by quantifying pathogen DNA at the different time points, which was successful in discriminating the genotypes. In other root pathosystems, such as barley/*Verticillium chlamydosporium*, the use of low temperature scanning electron microscopy (LTSEM) revealed details of the colonization process [49].

Steps of the pathogen life cycle corresponding to more advanced stages of disease development cycle would have been important to evaluate but could not be observed in this study due to a lack of adapted methodologies. Sporulation, measurable as oospore number, is a key life history trait, essential for pathogen multiplication. Kraft and Boge [27] measured sporulation by counting oospores on a Hawksley nematode-counting slide, from samples previously macerated in a Sorval microblender. Kjøller and Rosendahl [50] used stained roots with trypan blue and a microscope to evaluate oospore quantity in the roots. In our study, we attempted to extract oospores from the roots at seven and ten days after inoculation, for all the NILs in two blocks of experiment #3 (data not shown). Roots from five plants per pot were ground in a blender containing enzymes [51], then after maceration overnight, the suspension was vacuum filtrated and finally the oospores were counted on a Malassey blade. The results showed a low efficiency of oospore extraction. Thus further optimisation is required to improve the oospore extraction yield. Oospores viability, using a germination test [52], or zoospores attraction to root exudates [27] are also key pathogen life history traits that would be interesting to measure.

### Single QTL can act on one or both steps of the *A. euteiches* life cycle studied

To our knowledge, this is the first report of the effect of single resistance QTL on the disease development cycle of a root pathogen using NILs. Previously QTL effects were only described on aerial pathogen development [7, 9, 53]. In our study, significant effects of single major QTL on disease severity were consistent with those reported previously for controlled conditions using the same NILs and strains [25]. Significantly smaller QTL effects were also revealed using *A. euteiches* DNA quantification by Q-PCR. (i) The major-effect QTL *Ae-Ps7.6* (with PI180693 and 90–2131 resistance alleles) and *Ae-Ps4.5* (with 90–2079 resistance allele) significantly delayed symptom appearance and slowed down root colonization from the early stages even before symptoms appeared. The resistance allele originating from the pea line 552 at QTL *Ae-Ps7.6* only had a significant effect on reducing root colonization. Effects of resistance alleles at the two major QTL observed in the NILs were mostly consistent with their level of contribution to resistance as previously reported in RIL populations [19, 21]. (ii) The minor effect QTL *Ae-Ps2.2* (with PI180693 and 552 resistance alleles), *Ae-Ps3.1* (with 552 resistance allele) and *Ae-Ps5.1* (with 90–2131 resistance allele) significantly slowed down root colonization by *A. euteiches*, especially at later stages for QTL *Ae-Ps2.2* and *Ae-Ps3.1*. Resistance alleles from 552 at QTL *Ae-Ps2.2* and *AePs3.1*, as well as from 90–2131 at QTL *Ae-Ps5.1,* were previously detected with low effects for resistance to RB84 in RIL populations (R^2^ = 6.4-9.4 %; [19, 21]). In our pathosystem, symptom appearance and root colonization were expected to be independent, i.e. partial resistance could delay symptom appearance but not decrease pathogen colonization in the root or inversely, as previously observed [54]. However, in this study, we did not observe any pea lines that had an effect on delaying symptom appearance without decreasing root colonization. In some pathosystems, specific QTL with high effects on a single step of pathogen cycle, like the latency period [6, 9] or sporulation [10], were identified. However, several examples of individual QTL acting on several steps of disease development or pathogen life cycles [7, 8, 53, 55] have also been reported.

### Combinations of resistance QTL acting individually on similar steps of the disease development cycle can increase levels of partial resistance

Results from this study suggest that resistance QTL acting on similar steps of the disease development cycle could be pyramided to increase partial resistance efficiency. NILs carrying resistance alleles from 90–2131 at QTL *Ae-Ps5.1* and *Ae-Ps7.6,* both of which acted individually to limit root colonization by *A. euteiches*, showed a significantly increased effect on limiting the pathogen levels in roots and the disease severity, compared to NILs carrying each single QTL, particularly the major QTL *Ae-Ps7.6*. Cumulative QTL effects on limiting root colonization could also be observed for NILs carrying combinations of 552 resistance alleles at *Ae-Ps2.2, Ae-Ps3.1* and *Ae-Ps7.6*, but with a lower and non-significant level compared to single QTL effects. The resistant control lines, RIL 847.50 and 552, had significantly higher effects on reducing symptom appearance, root colonization by *A. euteiches* and disease severity progression compared to single QTL NILs. Similarly, a highly resistant pea line recently identified [20], AeD990SW45-8-7, was also evaluated and expressed much stronger effects than the multiple QTL NILs tested (*P* < 0.001; data not shown). Desgroux et al. [20] showed that the three lines AeD99OSW45-8-7, 552 and RIL 847.50, cumulated 12, nine and five favourable marker haplotypes, respectively, at 14 consistent Aphanomyces resistance loci detected by association genetics, including loci co-localizing with QTL *Ae-Ps1.2*, *Ae-Ps2.2*, *Ae-Ps3.1* and *Ae-Ps7.6* (Additional file 14 and Figure 2 in [20]). In our study, these four QTL all individually contributed to reduce the amount of pathogen DNA at one or more of the scoring dates. We could thus hypothesize that pyramiding resistance alleles at these four QTL, which appear to act especially by reducing *A. euteiches* root colonization, could lead to increased partial resistance levels.

However, this hypothesis does not exclude the possibility that the involvement of these QTL in other steps of the disease development or pathogen life cycle also contributes to increase resistance efficiency. Indeed, Kraft and Boge [27] showed that partial resistance to *A. euteiches* in the resistant germplasm PI180693 was associated with a slower development of symptoms and multiplication of the pathogen as revealed by ELISA. The authors also found reduced numbers of oospores and germinated zoospores in exudates from roots of PI180693. Resistance QTL apparently acting on similar steps of the pathogen life cycle could also be involved in different steps or molecular mechanisms controlling pathogenesis. Chung et al. [7] showed that a QTL controlling resistance to *Setosphaeria turcica* in maize enhanced the accumulation of callose and phenolics surrounding infection sites, reduced hyphal growth into the vascular bundle and impaired the subsequent necrotrophic colonization in the leaves.

## Conclusion

This study used NILs and Q-PCR-based pathogen DNA quantification to demonstrate that previously identified individual and combined resistance QTL delay symptom appearance and slow down pea root colonization by *A. euteiches*. Further method development would be required to investigate QTL effects on other steps of the pathogen life cycle, such as sporulation. Further work will also be necessary to validate whether the QTL effects observed under controlled conditions are also observed in infested fields. The durability of QTL combinations acting on similar steps of the disease development cycle will also have to be validated, as the pressure exerted by several QTL on a target phase of pathogen development may induce the appearance and/or the selection of isolates which may be able to overcome plant resistance efficient on this phase [56, 57].

## Abbreviations

AUDPC, area under the curve progression of the disease; CLMM, cumulative linked mixed model; CT, threshold cycle number; DNA, Deoxyribonucleic acid; DS, disease severity; DSP, dark skin perfection (germplasm); E, efficiency; ELISA, enzyme-linked immunoSorbent assay; GLMM, generalized linear mixed model; INRA, Institut National de la Recherche Agronomique; LMM, linear mixed model; LSMeans, least square means; MAB, marker-assisted backcrossing; NILs, near isogenic lines; Q-PCR, quantitative-polymerase chain reaction; QTL, quantitative trait loci; RILs, recombinant inbred lines; USA, United States of America.

## Additional files

Additional file 1: DS scores probability in each NIL set at each scoring day. **A** Experiments #1 and #2; **B** Experiments #3 and #4. The colors in each bar represent the probabilities of scores “0” (healthy plant) to “5” (dead plant), according to the DS rating scale used [25]. At two and three days after inoculation, no DS scores probability could be calculated from the CLMM model because some lines did not have symptoms. (PDF 1351 kb) Additional file 2: Effects of NILs carrying single or combined resistance on variables of the *Aphanomyces* root rot development cycle. A-C/ Single QTL NIL experiment #1; D/ Combined and single QTL NIL experiment #4; E/ Single QTL NIL experiment #2. The first graph represents the evolution of the probability of symptom appearance for seven days after inoculation, for each line. It corresponds to the percentage of plants with symptoms per block for each scoring day. The second graph shows for each line the root colonization speed, corresponding to the slope of the curve of pathogen DNA amounts per block, until 10 days after inoculation, from 10^4^ DNA copies detected. Pathogen DNA data was used from one biological replicate at the fourth day in experiments #1 and #2 and the seventh day in experiment #4. In the third graph, the AUDPC was calculated from the pathogen DNA quantification data over the ten days after inoculation. Bars represent standard errors. Attribution of each line to LSMeans group(s) is indicated by letter(s), according to the Tukey test (*P* < 0.05). Blue and red lines indicate the NIL without QTL and the donor or resistant control lines, respectively. (PDF 380 kb)
