# Supplementary material for: Single and multiple resistance QTL delay symptom appearance and slow down root colonization by Aphanomyces euteiches in pea near isogenic lines
Source: BMC Plant Biol. 2016 Jul 27;16:166. doi: 10.1186/s12870-016-0822-4 (PMC4964060; doi:10.1186/s12870-016-0822-4)

Additional file 1: DS scores probability in each NIL set at each scoring day

A Experiments #1 and #2. The colors in each bar represent the probabilities of scores "0" (healthy plant) to "5" (dead plant), according to the DS rating scale used [25]. At two and three days after inoculation, no DS scores probability could be calculated from the CLMM model because some lines did not have symptoms.

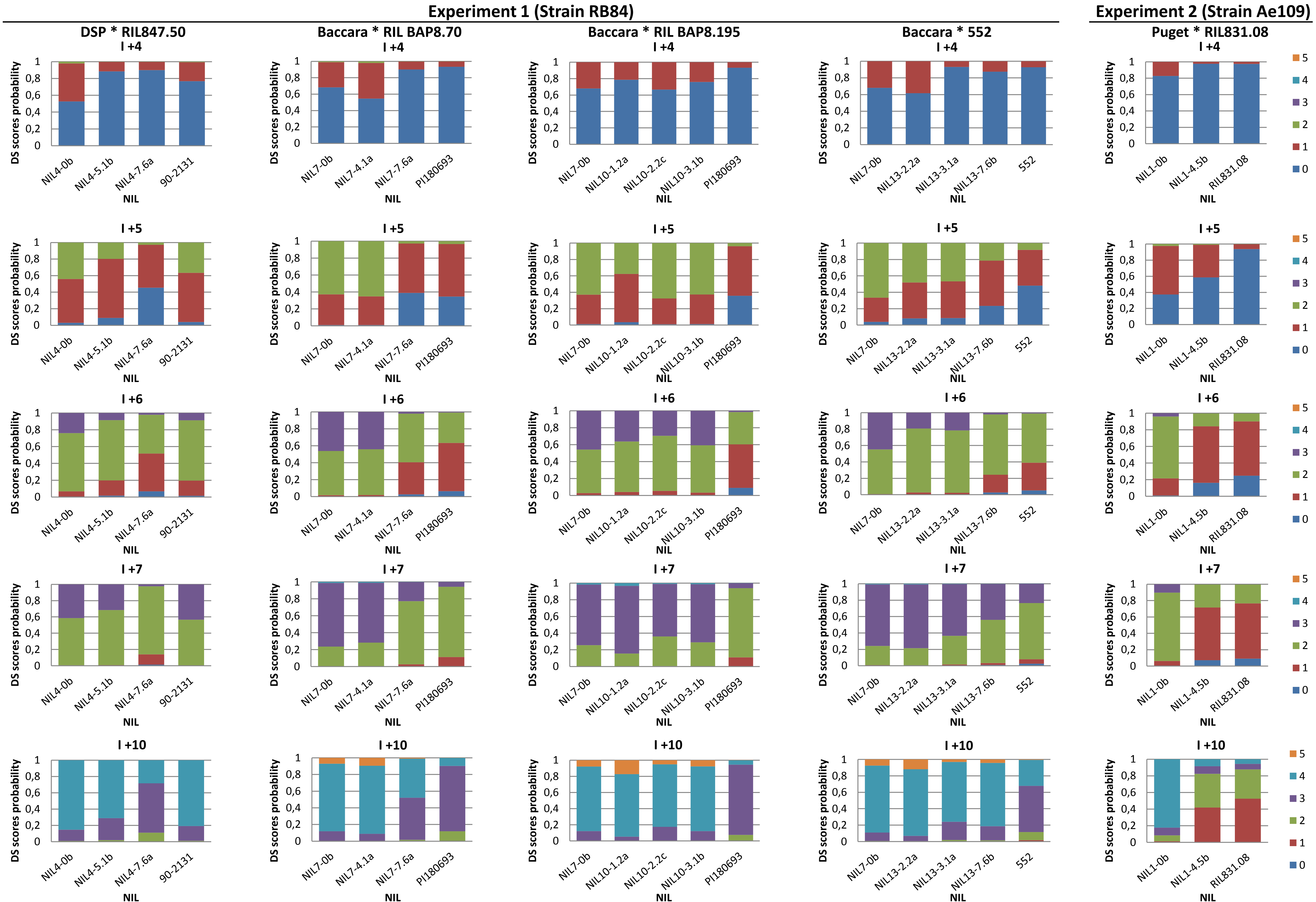

B Experiments #3 and #4. The colors in each bar represent the probabilities of scores “0” (healthy plant) to “5” (dead plant), according to the DS rating scale used [25]. At two and three days after inoculation, no DS scores probability could be calculated from the CLMM model because some lines did not have symptoms.

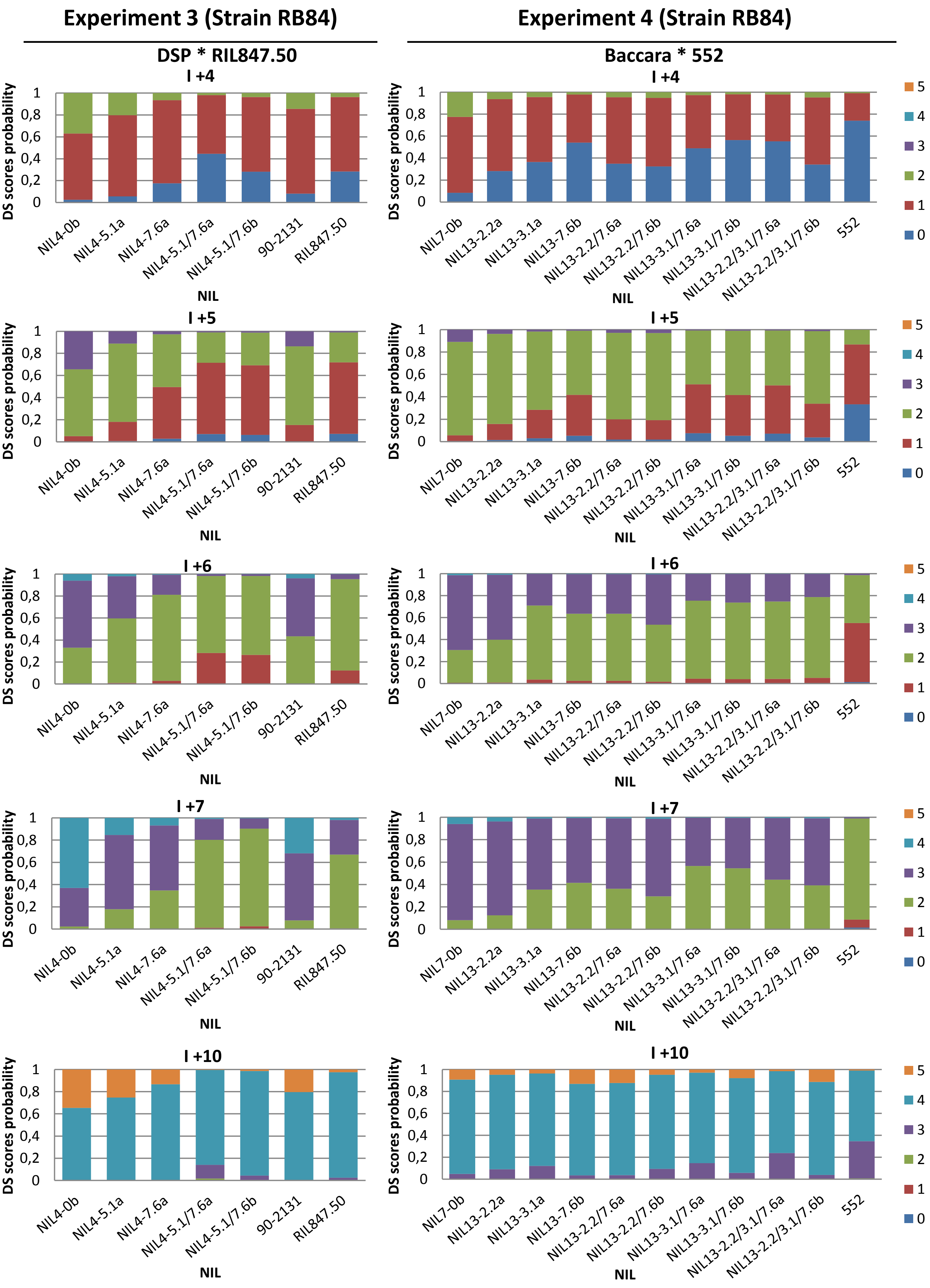

Supplement: Additional file 1: — DS scores probability in each NIL set at each scoring day. A Experiments #1 and #2; B Experiments #3 and #4. The colors in each bar represent the probabilities of scores “0” (healthy plant) to “5” (dead plant), according to the DS rating scale used [25]. At two and three days after inoculation, no DS scores probability could be calculated from the CLMM model because some lines did not have symptoms. (PDF 1351 kb) [file 12870_2016_822_MOESM1_ESM.pdf]
