# Supplementary material for: Single and multiple resistance QTL delay symptom appearance and slow down root colonization by Aphanomyces euteiches in pea near isogenic lines
Source: BMC Plant Biol. 2016 Jul 27;16:166. doi: 10.1186/s12870-016-0822-4 (PMC4964060; doi:10.1186/s12870-016-0822-4)

Additional file 2: Effects of NILs carrying single or combined resistance on variables of the *A. euteiches* life cycle.

A-C/ Single QTL NIL experiment #1; **D**/ Combined and single QTL NIL experiment #4; **E**/ Single QTL NIL experiment #2. The first graph represents the evolution of the probability of symptom appearance for seven days after inoculation, for each line. It corresponds to the percentage of plants with symptoms per block for each scoring day. The second graph shows for each line the root colonization speed, corresponding to the slope of the curve of pathogen DNA amounts per block, until 10 days after inoculation, from 10^4 DNA copies detected. In the third graph the AUDPC was calculated from the pathogen DNA quantification data over the ten days after inoculation. Bars represent standard errors. Attribution of each line to LSMeans group(s) is indicated by letter(s), according to the Tukey test (P<0.05). Blue and red lines indicate the NIL without QTL and the donor or resistant control lines, respectively.

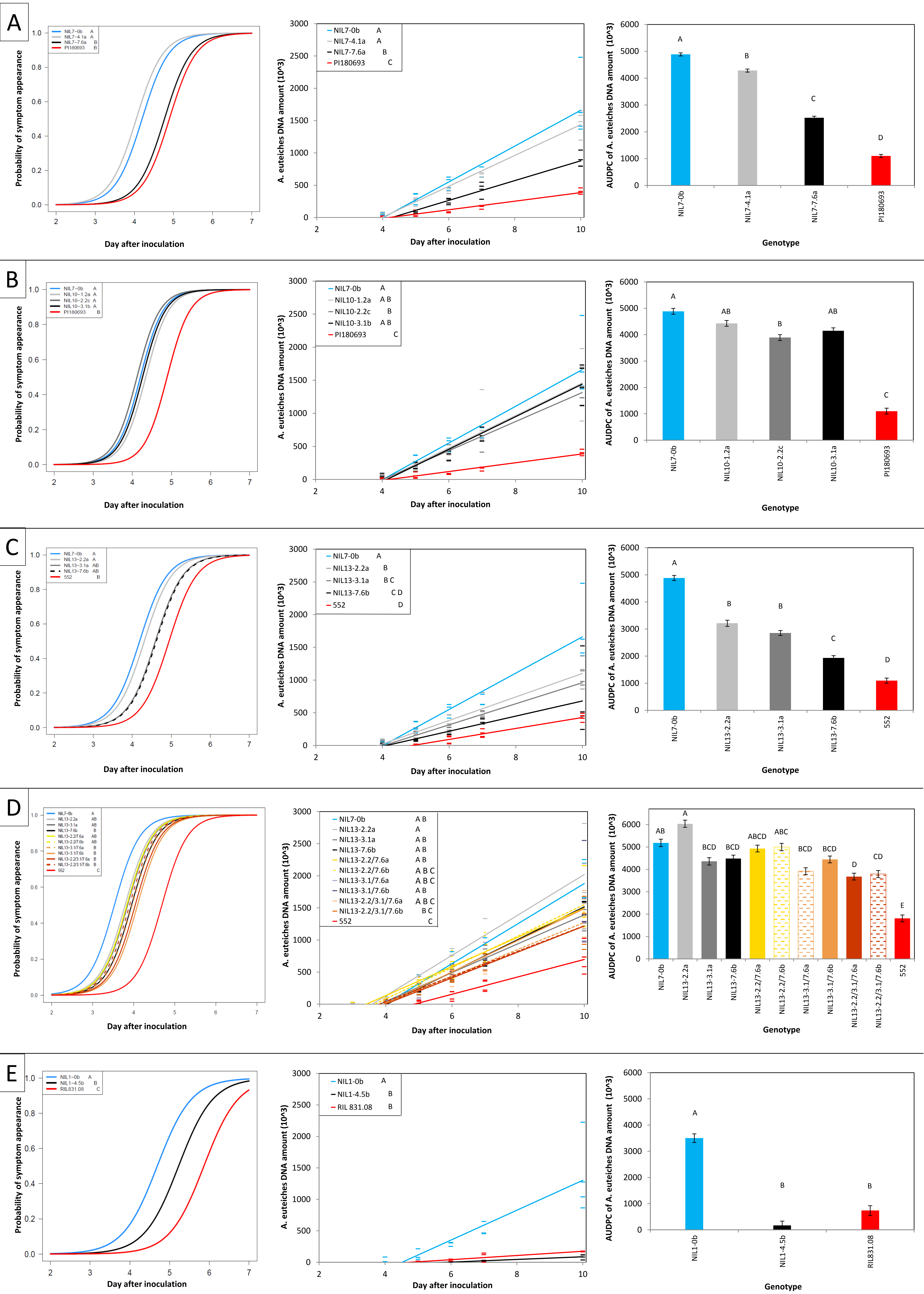

Supplement: Additional file 2: — Effects of NILs carrying single or combined resistance on variables of the Aphanomyces root rot development cycle. A-C/ Single QTL NIL experiment #1; D/ Combined and single QTL NIL experiment #4; E/ Single QTL NIL experiment #2. The first graph represents the evolution of the probability of symptom appearance for seven days after inoculation, for each line. It corresponds to the percentage of plants with symptoms per block for each scoring day. The second graph shows for each line the root colonization speed, corresponding to the slope of the curve of pathogen DNA amounts per block, until 10 days after inoculation, from 104 DNA copies detected. Pathogen DNA data was used from one biological replicate at the fourth day in experiments #1 and #2 and the seventh day in experiment #4. In the third graph, the AUDPC was calculated from the pathogen DNA quantification data over the ten days after inoculation. Bars represent standard errors. Attribution of each line to LSMeans group(s) is indicated by letter(s), according to the Tukey test (P < 0.05). Blue and red lines indicate the NIL without QTL and the donor or resistant control lines, respectively. (PDF 380 kb) [file 12870_2016_822_MOESM2_ESM.pdf]
